# The One Health European Joint Programme (OHEJP), 2018–2022: an exemplary One Health initiative

**DOI:** 10.1099/jmm.0.001228

**Published:** 2020-07-07

**Authors:** Helen L. Brown, Jade L. Passey, Maria Getino, Isabella Pursley, Piyali Basu, Daniel L. Horton, Roberto M. La Ragione

**Affiliations:** ^1^​ Department of Pathology and Infectious Diseases, School of Veterinary Medicine, University of Surrey, Daphne Jackson Road, Guildford, Surrey, GU2 7AL, UK; ^2^​ School of Dentistry, University of Cardiff, University Hospital Wales, Heath Park, Cardiff, CF14 4XY, UK

**Keywords:** One Health, Antimicrobial resistance, Zoonosis, Foodborne infections, Emerging threats

## Overview

One Health is an increasingly popular approach used to tackle complex health problems. The One Health concept recognizes that human health is tightly connected to the health of animals and the environment. Although the related fields are now more aware of the benefits of collaborative working, the full benefits have not yet been realized as research efforts are often focussed on just one of these health domains. To address regional and global issues such as foodborne zoonoses (FBZ), antimicrobial resistance (AMR) and emerging infectious threats (ET), there must be transdisciplinary collaboration between the health domains, in addition to active dialogue between scientists and international policy makers. This editorial introduces the One Health European Joint Programme (OHEJP) as an example of a One Health initiative.

### Zoonoses, AMR and their global burden

Zoonoses are infectious diseases that can be transmitted directly or indirectly between humans and animals. Although the severity of zoonotic infections varies, their global impact is undisputable. The World Bank estimates that just six zoonotic disease outbreaks between 1997 and 2009 led to a global economic loss of US$ 80 billion [1]. This high cost is due to medical costs, loss of individual productivity and restrictions on trade and movement during outbreaks. Despite improvements in the management and treatment of zoonotic outbreaks, high disease burdens caused by zoonotic pathogens continue to be reported globally. These problems have been amply demonstrated recently by the SARS-CoV-2 pandemic. Although it is still too soon to fully assess the total economic and societal cost of this virus, recent publications, such as Nicola *et al.* [2] have begun to highlight just how widespread the impact of a truly global zoonotic disease can be.

Alongside zoonoses, AMR is a growing international issue. The World Health Organization (WHO) has listed AMR as one of the ten greatest global health threats in 2019 [3]. AMR is defined as the ability of microorganisms to survive the effect of antimicrobial drugs, hindering not only our ability to treat infectious diseases, but also to perform medical procedures requiring prophylactic antibiotic administration. It has been predicted that by 2050, the number of deaths due to unresponsive infections will reach 10 million annually, with the associated costs being estimated at US$ 100 trillion [4]. Increased and inappropriate use of antimicrobials has contributed to the development and spread of AMR, which can be transmitted between humans, animals and the environment.

### The history of the ‘One Health’ concept

**Fig. 1.**
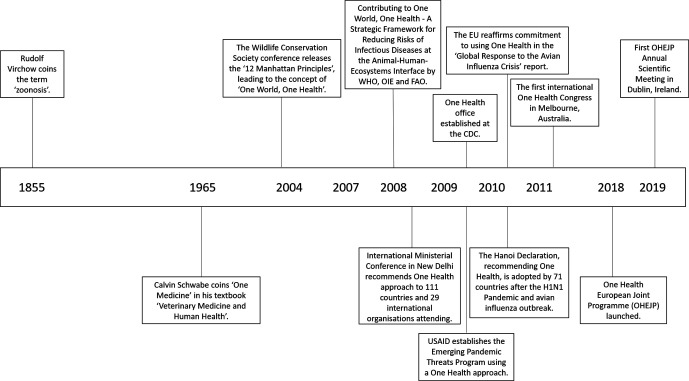
Timeline of selected key One Health milestones since 1855. An increasing uptake of One Health approaches by large agencies is evident from 2004 onwards.

The origins of One Health go as far back as 1855, when Rudolf Virchow founded comparative pathology, which could be seen as the origin of the One Health concept. Building upon this, Calvin W. Schwabe argued in the twentieth century against compartmentalization in medical research, using the term ‘One Medicine’. The term One Health was then popularized in 2004 by the Wildlife Conservation Society at a conference in New York [5], and its use has continued to evolve since then, fostering the revival of comparative medicine (Fig. 1, and reviewed in Gibbs [6]). One Health has now been adopted by the WHO [7], the Food and Agriculture Organization (FAO) [8] and the World Organization for Animal Health (OIE) [9].

### The importance of collaborative One Health projects

The use of a coordinated One Health approach has successfully tackled some zoonotic infections, although typically success is observed where problems are geographically restricted and controlled by a single governmental unit. One successful example involving multiple governments is the reduction in human *Salmonella* infections within the UK during the early 1990s. The infection was linked to the consumption of poultry products, in particular eggs [10]. Coordination between multiple government agencies and research agencies with expertise in human, animal and environmental health led to improved biosecurity on farms, surveillance, vaccination programmes for laying hens, and public health programmes [10]. This resulted in a nationwide food-safety scheme, the Lion Brand, which was introduced to highlight which eggs have been produced under the specified standards of food safety.

In contrast, AMR is still a global issue requiring One Health approaches to work on effective solutions [11]. Veterinarians, medical doctors, dentists, healthcare professionals and farmers have a significant role to play, allowing rapid detection and intervention at the source to reduce antibiotic use, particularly of critically important antibiotics. Despite the increasing uptake of the One Health approach to health issues, there are still measures to be taken to improve transdisciplinary collaboration of One Health research and surveillance to ensure approaches are harmonized for national and international benefit.

### From research outputs to policy changes

**Fig. 2.**
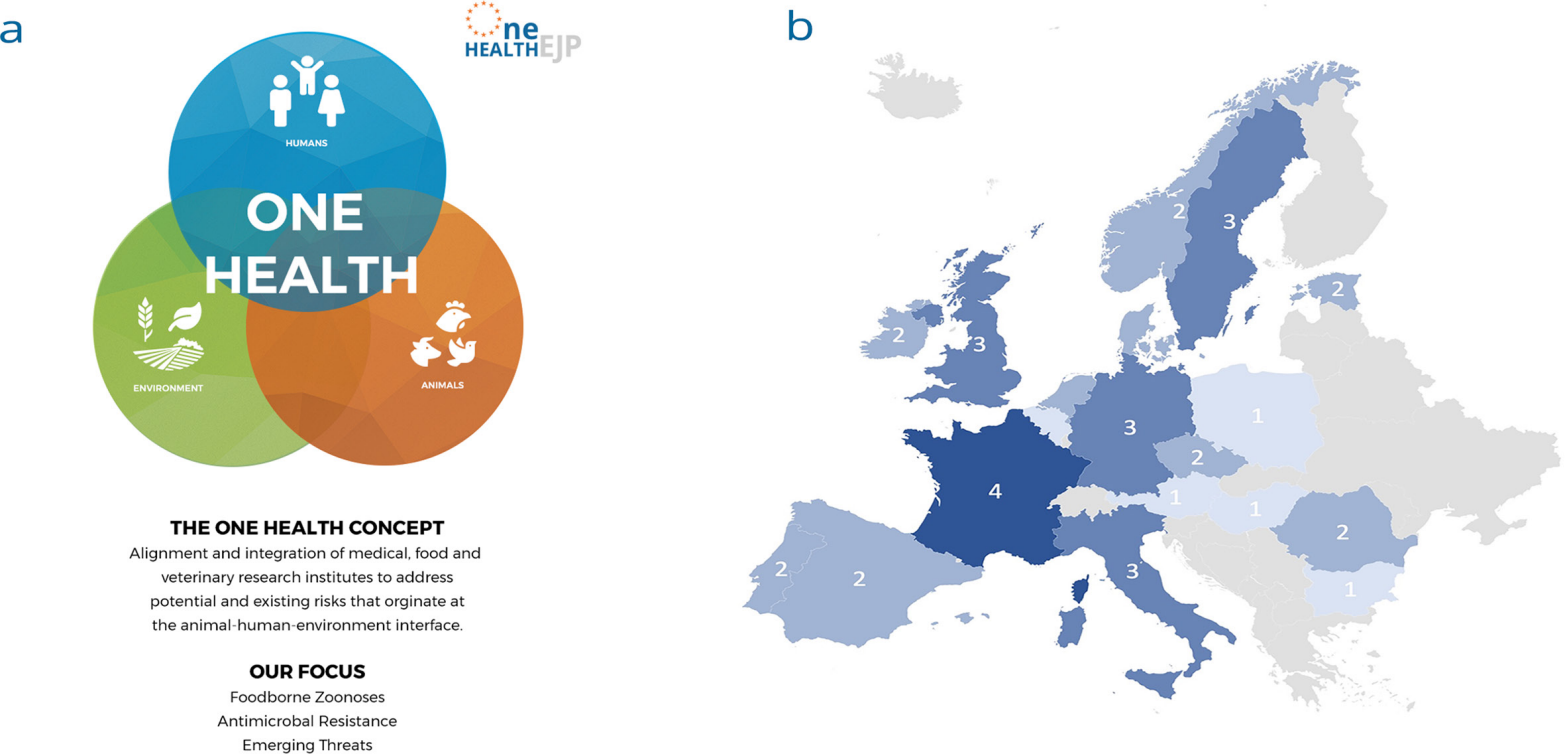
(a) Illustration of the One Health concept in the context of the One Health EJP, defining the key aim and focus of the programme. (b) Total number of partner institutes in each EU country within the One Health EJP consortium. For more information visit: www.onehealthejp.eu/consortium/

To address these challenges of interdisciplinary coordination, the five year OHEJP was established in 2018 and boasts a landmark partnership between 37 partners across 19 member states in Europe and the Med-Vet-Net-Association (Fig. 2). This programme is an interdisciplinary, collaborative and international approach to address One Health issues in the fields of FBZ, AMR and ET. Its main focus is to provide opportunities for the harmonization of approaches, methodologies, databases and procedures for the assessment and management of FBZ, AMR and ET across Europe, which will improve the quality and compatibility of information for decision making and informing policy change. The OHEJP has Joint Research Projects (JRP), Joint Integrative Projects (JIP), PhD projects, and it funds Short Term Missions each year. These are key instruments to facilitate partner institutes working together and aligning their approach, in addition to sharing their knowledge and expertise. Furthermore, the OHEJP aims to create a global ‘One Health Community’ through the research projects, PhD projects, workshops, Continuing Professional Development (CPD) modules, Summer Schools and Annual Scientific Meetings, which aim to attract both those inside and outside of the consortium to increase awareness and create opportunities for collaborative solutions.

The OHEJP has active dialogue with the key European agencies, the European Centre for Disease Prevention and Control (ECDC) and the European Food Safety Authority (EFSA) to ensure that One Health needs are addressed in a synergistic way. This approach aims to improve cross-disciplinary collaboration and communication, which in turn facilitates the OHEJP’s aim to translate science into policy and enables it to tackle FBZ, AMR and ET on a much larger scale. The OHEJP has a work package dedicated to translating science to policy and communicating with stakeholders, in particular with ECDC and EFSA, and international policy makers, including WHO, FAO and OIE. This communication is essential to identify and discuss research and integrative needs, and to ensure that the activities of the OHEJP meet these needs, e.g., by incorporating them into the OHEJP Strategic Research Agenda (found on our website at www.onehealthejp.eu), ensuring the results and outputs will be useful and utilized.

Furthermore, communication and dissemination are key components of the OHEJP. Effective communication will improve collaboration across sectors and the overall awareness of the general public, in addition to policy makers at all levels. The OHEJP aims to improve awareness of key issues through events, which attract audiences from across the globe, a website and social media networks. In summary, a globally coordinated approach is required, between the medical, veterinary and environmental professions to tackle emerging One Health issues, ultimately improving food security and human and animal health.
